# Implication of perineural invasion in patients with stage II gastric cancer

**DOI:** 10.1186/s12957-023-03236-x

**Published:** 2023-11-29

**Authors:** Dandong Luo, Yue-e Wen, Huaxian Chen, Zijian Deng, Jiabo Zheng, Shi Chen, Junsheng Peng, Lei Lian

**Affiliations:** 1 Department of General Surgery (Department of Gastric Surgery), The Sixth Affiliated Hospital, Sun Yat-Sen University, 26 Yuancun Er Heng Rd. Guangzhou, Guangdong, China; 2 Guangdong Provincial Key Laboratory of Colorectal and Pelvic Floor Diseases, The Sixth Affiliated Hospital, Sun Yat-Sen University, Guangzhou, China; 3 Biomedical Innovation Center, The Sixth Affiliated Hospital, Sun Yat-Sen University, Guangzhou, China; 4 Department of Pathology, The First People’s Hospital of Kashi Prefecture, Kashi, China

**Keywords:** Stage II gastric cancer, Perineural invasion, Adjuvant chemotherapy, Prognosis

## Abstract

**Background:**

Perineural invasion (PNI) is regarded as a prognostic factor for patients with GC. However, the significance of PNI in patients with stage II GC remains unclear. This study aimed to investigate the clinical implication of PNI in patients with stage II GC undergoing curative resection.

**Methods:**

Patients with stage II GC who underwent curative resection were retrospectively evaluated from January 2010 to July 2019. According to PNI status, all patients were divided into two groups: with or without PNI. The prognostic value of PNI was analyzed by univariate and multivariate Cox proportional hazards regression models.

**Results:**

A total of 233 patients were included in this study. There were 100 patients with PNI (42.92%) and 133 patients without PNI (57.08%). The overall survival (OS) and disease-free survival (DFS) rates for patients with PNI were significantly lower than that for patients without PNI (*p* = 0.019 and *p* = 0.032, respectively). Multivariate analysis indicated that the presence of PNI was an independent risk factor for OS (hazard ratio (HR): 1.76, 95% confidence interval (CI) 1.02–3.06, *p* = 0.044) and DFS (HR: 1.70, 95% CI 1.04–2.80, *p* = 0.035), while adjuvant chemotherapy (AC) was an independent protective factor for OS (HR: 0.51, 95% CI 0.30–0.88, *p* = 0.016) and DFS (HR: 0.52, 95% CI 0.31–0.86, *p* = 0.011). Furthermore, among patients with PNI, those who received AC had better OS (*p* = 0.022) and DFS (*p* = 0.027) than their counterparts. When patients with PNI received AC, the OS (*p* = 0.603) and DFS (*p* = 0.745) appeared to be similar to those without PNI and no AC.

**Conclusion:**

In patients with stage II GC undergoing curative resection, the presence of PNI was associated with worse survival, which appeared to improve with the treatment of AC, indicating a potential need for more intensive AC.

**Supplementary Information:**

The online version contains supplementary material available at 10.1186/s12957-023-03236-x.

## Introduction

Gastric cancer (GC) is the third leading cause of cancer-related death worldwide [1]. The 5-year survival rate for GC is not satisfactory because over 80% of patients present with advanced disease in China [2]. Stage II GC accounts for about 20% of all GC patients [3], and radical surgical resection combined with D2 lymph node dissection is the favored treatment strategy. Although the 5-year survival rate ranges from 68.3% to 75.6% in patients with stage II GC undergoing radical resection, approximately 30% of patients eventually develop recurrence and metastasis [4–6]. In CLASSIC trial, patients with stage II or IIIB GC showed a significantly better 5-year DFS with postoperative chemotherapy than with surgery alone [7, 8]. At present, adjuvant chemotherapy (AC) has been recommended as standard of care for stage II GC [9–11].

The prognosis of stage II GC patients may vary significantly, and AC may not provide additional benefits for all patients but rather carry the risk of potential adverse side effects. A previous study has reported that lymphatic invasion was an independent risk factor for T3N0 GC and suggested that T3N0 GC patients with lymphatic invasion may benefit from AC [12]. However, AC cannot improve the prognosis of elderly patients with stage II GC [4]. Therefore, further stratified studies are needed to investigate useful biomarkers to distinguish individual subgroups of patients with stage II GC who may benefit from AC.

Perineural invasion (PNI) is a pathological characteristic indicating the infiltration of tumor cells along the perineurium or the neural fascicle and representing more aggressive biological behaviour of tumor cells [13]. Several studies have demonstrated that PNI was one of the independent factors associated with early recurrence and poorer survival in GC patients after D2 gastroenterectomy [13–15]. Studies have reported that T2N0 GC patients with PNI may benefit from AC [16]. According to a study by Qing Tao et al., PNI might be an independent predictor for the efficacy of AC in stage Ib-III GC patients with radical resection, but further subgroup analysis was not performed. Therefore, whether PNI can be predictive of the benefit of AC in stage II GC patients is still unclear.

Therefore, we conducted a retrospective study to investigate the association between PNI and clinicopathological features and determine the prognostic impact of PNI in patients with stage II GC.

## Methods

### Patient selection

**Fig. 1:**
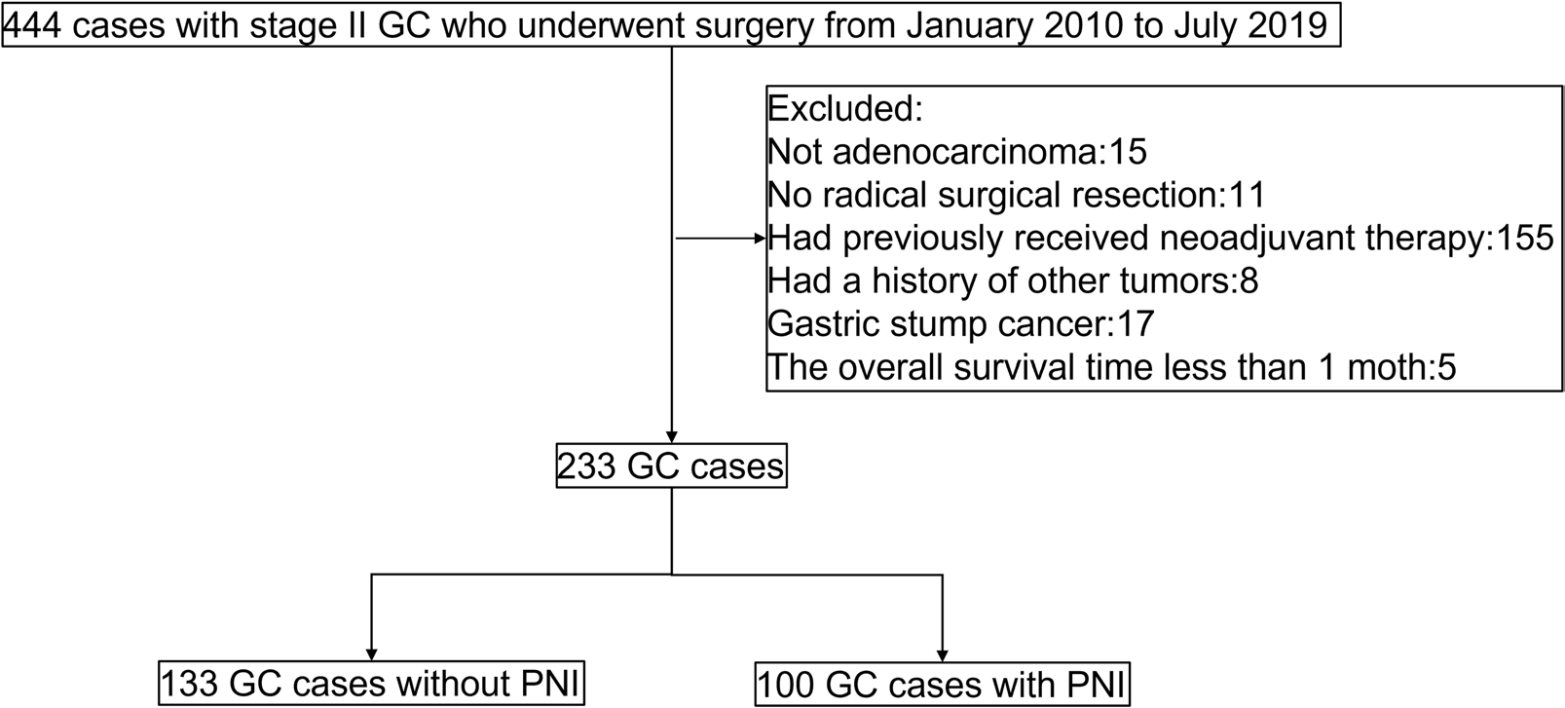
Flow chart of the study

Patients with stage II GC who underwent curative resection in the Sixth Affiliated Hospital of Sun Yat-sen University from January 2010 to July 2019 were retrospectively analyzed. This study was approved by the Ethics Committee of the Sixth Affiliated Hospital of Sun Yat-sen University (No. 2021ZSLYEC-325). The inclusion criteria were as follows: (1) patients pathologically diagnosed with stage II gastric adenocarcinoma; (2) initial diagnosis and treatment-naïve; and (3) underwent D2 radical gastrectomy with R0 resection. Patients were excluded if any of the following was present: (1) incomplete or missing clinical and/or follow-up data; (2) a history of any other malignant tumors; (3) other types of GC except gastric adenocarcinoma; (4) gastric stump tumor; (5) underwent neoadjuvant therapy before radical surgery; and (6) overall survival (OS) time < 30 days. A flowchart of patient selection is shown in Fig. 1.

### Data collection

Clinicopathological data were obtained from a prospectively maintained GC Database. The location of GC was divided into four regions: upper, middle, lower, and gastroesophageal junction (EGJ). The type of gastrectomy included total or subtotal gastrectomy. The preoperative carcinoma embryonic antigen (CEA) level recorded was the result of the most recent test conducted before the operation. Pathological features include histological type, histological differentiation grade, Lauren classification, lymph node metastasis, tumor node metastasis (TNM) stage, vascular invasion, and PNI. The histological differentiation types of GC were divided into well differentiation (well differentiated adenocarcinoma and moderately differentiated adenocarcinoma) and poor differentiation (poorly differentiated adenocarcinoma, mucinous adenocarcinoma, and signet ring cell carcinoma). Lauren classification were categorized according to the Lauren criteria [17]. TNM stage was determined according to the guidelines of the American Joint Committee on Cancer TNM staging system (Version 8th) [16]. After surgery, part of the patients were treated with 5-fluorouracil-based AC. AC was defined as at least four cycle of 5-fluorouracil-based chemotherapy [18]. The primary outcomes were OS and disease-free survival (DFS). OS is defined as the time from the date of curative surgery of GC to the date of death from any cause or until the last contact. DFS is defined as the time from the date of curative surgery of GC to the date of a patient's recurrence or death due to any cause. ​The recurrence event were according to previous study [19].

### Statistical method

Statistical evaluation was executed using R software (http://www.r-project.org, version 4.1.2). Continuous variables were tested for normal distribution using the Shapiro–Wilk normality test. Continuous variables with normal distribution are presented as mean (standard deviation), and continuous variables with nonnormal distribution are presented as median (interquartile range). The Mann–Whitney U test or independent sample t-test was used for continuous variables. For categorical variables, Pearson's *Chi*-squared test or Fisher's exact test was performed. After the *Chi*-squared test, post-hoc analysis was carried out using the Bonferroni correction. Patient prognosis was evaluated through OS and DFS. Survival was estimated using the Kaplan–Meier method, and the difference in survival rates between subgroups was tested for statistical significance with log-rank test. The prognostic factors for OS and DFS were assessed using univariate and multivariate Cox regression analyses. The variables with *p* values < 0.1 in the univariate analyses were included in the multivariate Cox regression analyses. All statistical tests were two-sided, and a *p* value < 0.05 was considered statistically significant.

## Results

### The clinicopathological characteristic of the whole cohort

​A total of 233 eligible patients with stage II GC were included in this study (Fig. 1), and the clinical and pathological characteristics of all patients are summarized in Table 1. There were 75 women (32.19%) and 158 men (67.81%). The mean age was (59.04 ± 12.68) years old (range, 21–89 years). One hundred and nine (46.78%) patients underwent total gastrectomy, and 124 (53.22%) patients underwent partial gastrectomy. AC was administered in 158 (67.81%) patients. On pathological examination, 135 (57.94%) patients had stage IIA disease, and 98 (42.06%) patients had stage IIB disease. There were 100 (42.92%) patients with PNI and 133 (57.08%) patients without PNI. In addition, vascular invasion was present in 50 (21.46%) patients, and lymph node metastasis was present in 114 (48.93%). The median follow-up time was 51.0 months. During follow-up, 65 (27.90%) patients relapsed or deceased. The OS were 97.0%, 83.3%, and 73.1% at 1, 3, and 5 years, respectively, while the DFS were 95.7%, 80.8%, and 67.5% at 1, 3, and 5 years, respectively.

**Table 1:** Relationship between PNI and clinicopathological features in stage II GC

Characteristics		PNI		*p* value
		Absence (*N* = 133)	Presence (*N* = 100)	
Gender (%)	Female	40 (30.08)	35 (35)	0.513
	Male	93 (69.92)	65 (65)	
Age (mean ± SD, years)		60.81 ± 11.94	56.69 ± 13.29	0.014
Age (%)	< 60 years	56 (42.11)	55 (55.00)	0.069
	≥ 60 years	77 (57.89)	45 (45.00)	
Tumor location (%)	Lower	67 (50.38)	53 (53)	0.305
	Middle	18 (13.53)	19 (19)	
	Upper	29 (21.80)	13 (13)	
	EGJ	19 (14.29)	15 (15)	
Type of gastrectomy (%)	Partial	72 (54.14)	52 (52)	0.849
	Total	61 (45.86)	48 (48)	
Adjuvant chemotherapy (%)	No	44 (33.08)	31 (31)	0.845
	Yes	89 (66.92)	69 (69)	
CEA (ng/ml) (%)	≤ 5	112 (84.21)	87 (87)	0.682
	> 5	21 (15.79)	13 (13)	
Vascular invasion (%)	Absence	105 (78.95)	78 (78)	0.99
	Presence	28 (21.05)	22 (22)	
Differentiation type (%)	Well differentiated	46 (34.59)	14 (14)	< 0.001
	Poorly differentiated	87 (65.41)	86 (86)	
Lauren classification (%)	Intestinal	49 (36.84)	16 (16)	0.005
	Diffuse	44 (33.08)	49 (49)	
	Mix	32 (24.06)	28 (28)	
	Others	8 (6.02)	7 (7)	
Tumor diameter (cm) (mean ± SD)		4.02 ± 2.18	4.02 ± 2.13	0.999
Tumor diameter (cm) (%)	≤ 3	60 (45.11)	41 (41)	0.622
	> 3	73 (54.89)	59 (59)	
T Stage (%)	T1	9 (6.77)	0 (0)	< 0.001
	T2	28 (21.05)	5 (5)	
	T3	95 (71.43)	86 (86)	
	T4a	1 (0.75)	9 (9)	
Lymph node metastasis (%)	Absence	60 (45.11)	59 (59)	0.049
	Presence	73 (54.89)	41 (41)	
TNM Stage (%)	IIA	83 (62.41)	52 (52)	0.145
	IIB	50 (37.59)	48 (48)	
Overall survival (%)	Alive	107 (80.45)	69 (69)	0.063
	Dead	26 (19.55)	31 (31)	
Disease-free survival (%)	No recurrence	102 (76.69)	66 (66)	0.098
	Recurrence or death	31 (23.31)	34 (34)	

*CEA* carcinoembryonic antigen, *SD* standard deviation, *EGJ* esophagogastric junction

### Association between PNI and clinical pathological features

Compared with patients without PNI, patients with PNI were younger and were more likely to have more advanced T stage, poor tumor differentiation, lower rate of lymph node metastasis, and a higher proportion of diffuse type or mixed type (*p* < 0.05) by Lauren’s classification. There were no differences between the two groups in tumor location, CEA, tumor diameter, vascular invasion, and TNM stage (*p* > 0.05). Types of procedure and use of AC were similar (*p* > 0.05). Patients with PNI were more likely to develop peritoneal metastases (52.94% vs 16.13%, *P* = 0.038, Supplementary Table1).

### Comparison of OS and DFS between different PNI groups

**Fig. 2:**
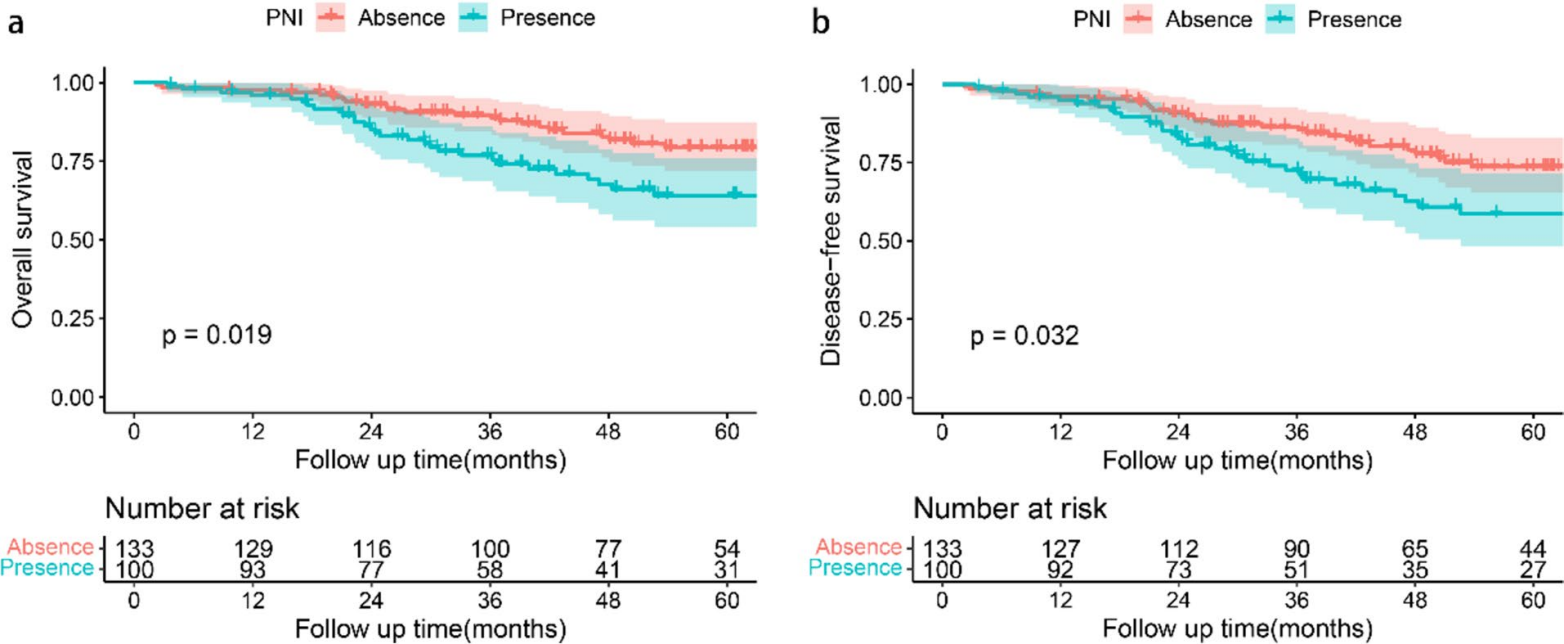
Survival curve analysis showed that the patients with PNI had worse OS (**a**. *p* = 0.019) and DFS (**b**. *p* = 0.032) than those without PNI

Kaplan–Meier curve analysis was performed to explore the role of PNI in patients with stage II GC. The OS and DFS for patients with PNI were significantly lower than that for patients without PNI (*p* = 0.019 and *p* = 0.032, respectively) (Fig. 2).

### Univariate and multivariate analyses for OS and DFS

According to univariate analysis, age, CEA, PNI, tumor location, AC, and T stage were associated with OS in patients with stage II GC (Table 2). The variables for the multivariate analysis were selected from the univariate analysis when *p* < 0.1. Multivariate analysis showed that CEA > 5 ng/mL (HR = 2.20, 95% CI 1.17–4.17, *p* = 0.015), the presence of PNI (HR = 1.76, 95% CI 1.02–3.06, *p* = 0.044), and tumor located in the middle stomach (HR = 3.19, 95% CI 1.62–6.26, *p* < 0.001) were associated with poor OS, while AC (HR = 0.51, 95% CI 0.30–0.88, *p* = 0.016) was associated with better OS (Table 2).

**Table 2:** Univariate and Multivariate analysis for predictors of overall survival

Characteristics	Univariate		Multivariate	
	HR (95%CI)	*p* value	HR (95%CI)	*p* value
Gender (Male)	1.41(0.77–2.57)	0.267		
Age (≥ 60 years)	1.44(0.84–2.46)	0.180		
CEA (> 5 ng/ml)	1.93(1.04–3.59)	0.037	2.20(1.17—4.17)	0.015
PNI (Presence)	1.85(1.1–3.13)	0.021	1.76(1.02 – 3.06)	0.044
Vascular invasion (Presence)	1.32(0.73–2.4)	0.352		
Location (Ref: Lower)				
Middle	2.87(1.48–5.53)	0.002	3.19(1.62 – 6.26)	< 0.001
Upper	1.04(0.48–2.25)	0.918	0.97(0.44—2.12)	0.931
EGJ	1.59(0.76–3.35)	0.221	1.58(0.75—3.33)	0.233
Tumor diameter (> 3 cm)	0.75(0.45–1.27)	0.290		
Type of gastrectomy (Total)	1.20(0.72–2.03)	0.482		
Adjuvant chemotherapy (Yes)	0.59(0.35–1.01)	0.055	0.51(0.30—0.88)	0.016
Differentiation type (Poorly differentiated)	1.25(0.66–2.37)	0.490		
Lauren classification (Ref: Intestinal)				
Diffuse	1.4(0.73–2.68)	0.316		
Mix	1.15(0.55–2.41)	0.718		
Others	0.5(0.13–1.91)	0.309		
T Stage (T3/T4a)	2.33(0.93–5.83)	0.072	1.55(0.59 – 4.03)	0.371
Lymph node metastasis (Presence)	1.3(0.77–2.19)	0.329		

*CEA* carcinoembryonic antigen, *PNI* perineural invasion, *EGJ* esophagogastric junction, *HR* hazard ratio, *CI* confidence interval

In addition, Cox regression analysis was performed to determine the potential value of PNI for DFS. Multivariate analysis indicated that the presence of PNI (HR = 1.70, 95% CI 1.04–2.80, *p* = 0.035), middle of the stomach (HR = 3.26, 95% CI 1.73–6.14, *p* < 0.001) and CEA > 5 ng/ml (HR = 2.34, 95% CI 1.30—4.21, *p* = 0.005) were associated with poor DFS, while AC (HR = 0.52, 95% CI 0.31–0.86, *p* = 0.011) was associated with better DFS (Table 3).

**Table 3:** Univariate and Multivariate analysis for predictors of disease-free survival

Characteristics	Univariate		Multivariate	
	HR (95%CI)	*p* value	HR (95%CI)	*p* value
Gender (Male)	1.45(0.82–2.55)	0.198		
Age (≥ 60 years)	1.28(0.78–2.09)	0.333		
CEA (> 5 ng/ml)	2.01(1.13–3.59)	0.018	2.34(1.30 – 4.21)	0.005
PNI (presence)	1.7(1.04–2.77)	0.034	1.70(1.04 – 2.8)	0.035
Vascular invasion (Presence)	1.16(0.66–2.05)	0.604		
Location (Ref: Lower)				
Middle	2.86(1.54–5.31)	< 0.001	3.26(1.73 – 6.14)	< 0.001
Upper	0.96(0.47–1.99)	0.916	0.96(0.46—1.99)	0.904
EGJ	1.48(0.73–2.99)	0.273	1.57(0.77—3.18)	0.211
Tumor diameter (> 3 cm)	0.73(0.45–1.18)	0.200		
Type of gastrectomy (Total)	1.2(0.74–1.95)	0.460		
Adjuvant chemotherapy (Yes)	0.6(0.36–1)	0.048	0.52(0.31—0.86)	0.011
Differentiation type (Poorly differentiated)	1.11(0.62–1.98)	0.727		
Lauren classification (Ref: Intestinal)				
Diffuse	1.29(0.71–2.36)	0.402		
Mix	1.1(0.55–2.19)	0.781		
Others	0.39(0.1–1.45)	0.159		
T Stage (T3/T4a)	1.66(0.76–3.65)	0.205		
Lymph node metastasis (presence)	1.33(0.81–2.16)	0.258		

*CEA* carcinoembryonic antigen, *PNI* perineural invasion, *EGJ* esophagogastric junction, *HR* hazard ratio, *CI* confidence interval

### Correlation between PNI and benefit of AC

**Fig. 3:**
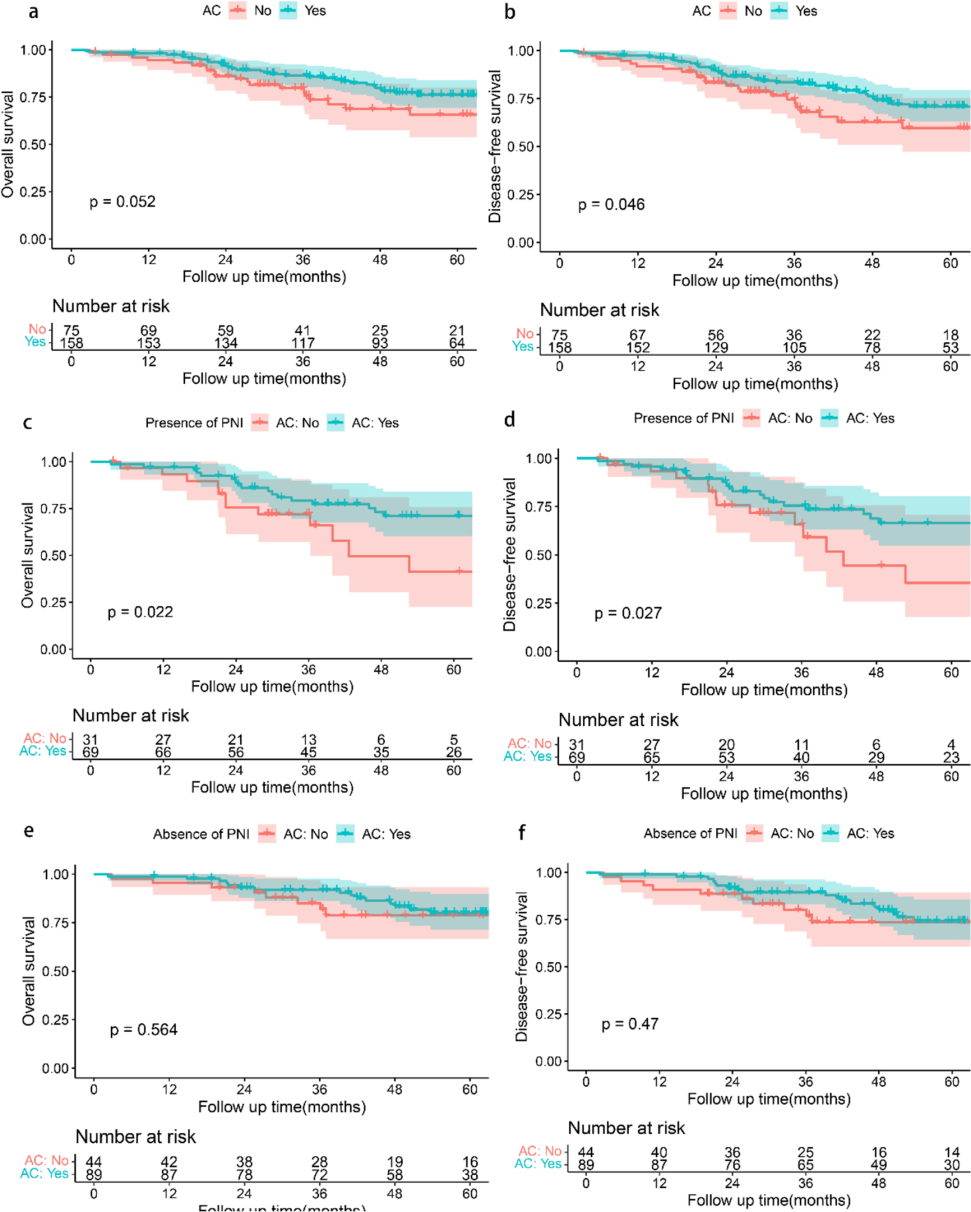
Survival curve analysis showed that patients undergoing AC had similar OS (**a**. *p* = 0.052) and better DFS (**b**. *p* = 0.046) than those who did not undergo AC. For the patients with PNI, the patients who underwent AC had better OS (**c**. *p* = 0.022) and DFS (**d**. *p* = 0.027) than those who did not undergo AC. For the patients without PNI, the OS (**e**. *p* = 0.564) and DFS (**f**. *p* = 0.47) of patients with PNI and who did not undergo AC were similar

**Fig. 4:**
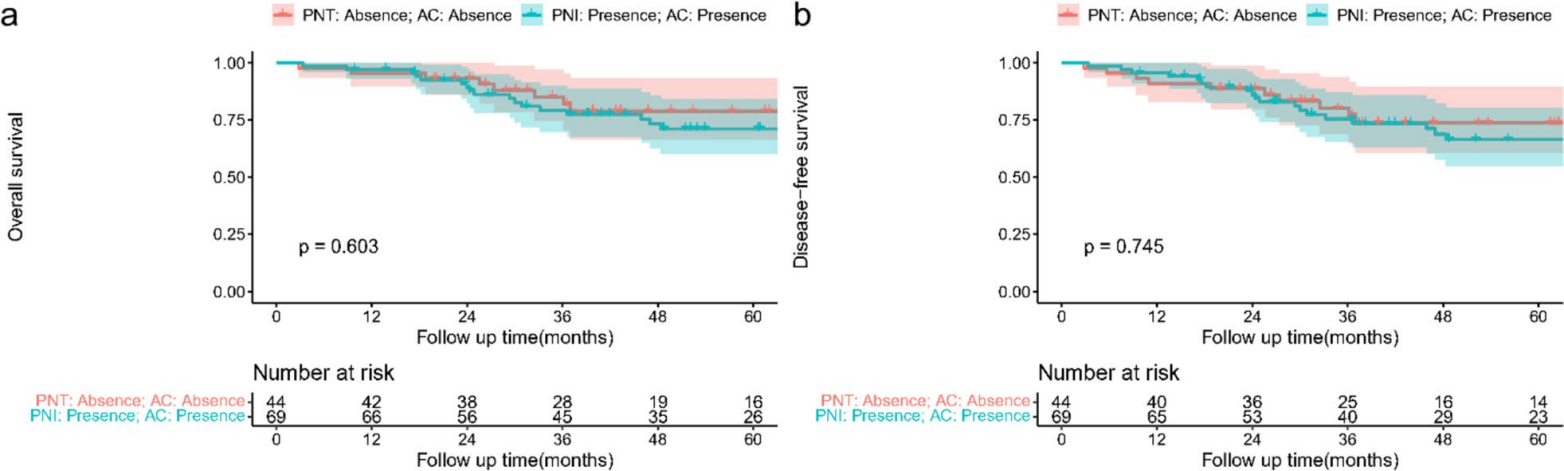
Survival curve analysis showed that patients with PNI and undergoing AC had similar OS (**a**. *p* = 0.603) and DFS (**b**. *p* = 0.745) to those absence of PNI and no AC

Among patients with stage II GC, patients who received AC had a better prognosis for DFS than those who did not (*p* = 0.046, Fig. 3b), but there was no difference between the two groups in OS (*p* = 0.052, Fig. 3a). Therefore, we analyzed whether patients with PNI or without PNI could benefit from AC. Among the patients with PNI, patients who received AC had better OS than those who did not receive AC (*p* = 0.022, Fig. 3c) and DFS(*p* = 0.027, Fig. 3d).​ Among patients without PNI, there was no significant difference between patients who received AC and those who did not in OS (*p* = 0.564, Fig. 3e) and DFS (*p* = 0.470, Fig. 3f), which means patients without PNI were associated with well prognosis. Of note, patients with PNI and receiving AC had a similar OS (*p* = 0.603, Fig. 4a) and DFS (*p* = 0.745, Fig. 4b) to those who did not receive AC and in the absence of PNI.

## Discussion

In the present study, we assessed the relationships between PNI and the clinical features and investigated the potential prognostic value of PNI in patients with stage II GC undergoing radical resection. We found that the presence of PNI was associated with several factors including advanced T stage, undifferentiated type, and higher proportion of diffuse type or mixed type by Lauren’s classification. The results also showed that the presence of PNI was an independent risk factor in patients with stage II GC who underwent radical surgical resection. Finally, patients with PNI and receiving AC had a similar prognosis to those who did not receive AC and in the absence of PNI.

PNI is regarded as a prognostic factor for patients with GC. Studies have reported that PNI could trigger cholinergic signals and lead to promoted tumor growth by inducing an immunosuppressive microenvironment characterized by impaired CD8 + T cell infiltration and a reduced Th1/Th2 ratio [20]. Research has indicated that GC cells exhibiting elevated expression of VACM1 have the capacity to stimulate the proliferation of progenitor cells and the outgrowth of neurites. These processes, in turn, contribute to the augmentation of tumor migration and the promotion of PNI [21]. In the context of the tumor immune microenvironment, it has been observed that PNI-negative tumors tend to exhibit higher levels of hypoxia compared to PNI-positive tumors. Furthermore, PNI-negative tumors demonstrate a relative upregulation in signaling pathways that are crucial for 5-FU metabolism or resistance [14]. Furthermore, PNI is associated with more aggressive biological behavior and strongly associated with increased tumor recurrence and worse survival in many types of cancer [22]. Our results also showed that PNI was related to undifferentiated and Lauren diffuse or mixed type, and advanced T stage in stage II GC. According to previous studies, PNI was a predictive factor for survival in head and neck, prostate, and colorectal cancer patients who underwent surgical resection [23, 24]. Another study also reported that the presence of PNI is an independent risk prognostic factor of DFS and cancer-specific survival of stage II/III GC [25]. For stage II GC after radical gastrectomy, PNI showed a predictive value for recurrence [26]. Similar with the literature, our results showed that the presence of PNI had a higher risk of recurrence and shorter OS in the patients with stage II GC. Therefore, PNI may be a useful and robust biomarker for prognosis in patients with stage II GC.

In order to reduce the risk of recurrence and improve the prognosis of GC, AC has been recommended as the standard postoperative treatment for advanced GC [27]. However, the role of AC for patients with stage II GC remains controversial [28, 29]. The JCOG1104 study, a randomized phase III trial, showed that the patients with stage II GC who received radical gastrectomy should receive eight cycle S1 AC for a more favorable RFS [11]. However, Zheng et al*.* reported that AC is not a prognostic factor for stage II GC [26]. PNI has been shown to be a valuable prognostic factor, so whether PNI can serve as a biomarker to assist decision-making for AC in stage II GC is still unknown. Our study found that stage II GC patients with PNI who received AC after surgery had significantly better OS and less recurrence. These results echo previous studies, which showed that a minimum of 3 cycles of postoperative AC in pT3N0M0 GC patients with PNI significantly reduced overall recurrence rate [30]. Xiao et al*.* found that AC improved OS and DFS in stage IB-III GC patients with PNI [14]. However, whether stage II GC with PNI needs AC has not been discussed. The patients with PNI who received AC had better survival and lower risk of recurrence.

There are some limitations in this study. First, it is a retrospective study with a small sample size. Secondly, the patients in our study cohort were not treated with radiotherapy. Finally, use of chemotherapy was decided at the discretion of both attending physician and patients. Therefore, our findings may be biased and further prospective randomized trials is warranted.

## Conclusion

In patients with stage II GC undergoing curative resection, the presence of PNI was associated with worse survival, which appeared to improve with the treatment of AC, indicating a potential need for more intensive AC.

### Supplementary Information

**Additional file 1: Supplementary Figure 1.** Survival curve analysis showed whether patients with or without PNI received adjuvant chemotherapy (A) or without adjuvant chemotherapy (B). **Supplementary Table1.** Recurrence pattern of Stage II GC patients with PNI. **Supplementary Table2.** T stage and Lymph node metastasis of Stage II GC patients with or without PNI

## Data Availability

All analyzed data are included in this published article. The original data are available upon reasonable request to the corresponding author.

## Abbreviations

AC: Adjuvant chemotherapy
CEA: Carcinoma embryonic antigen
CI: Confidence interval
DFS: Disease-free survival
EGJ: Esophagogastric junction
GC: Gastric cancer
HR: Hazard ratio
OS: Overall survival
PNI: Perineural invasion
TNM: Tumor node metastasis

## References

[1] Sung H, Ferlay J, Siegel RL (2021). Global Cancer Statistics 2020: GLOBOCAN Estimates of Incidence and Mortality Worldwide for 36 Cancers in 185 Countries. CA Cancer J Clin.

[2] Chen Z, Liu Z, Huang W (2017). Gimatecan exerts potent antitumor activity against gastric cancer in vitro and in vivo via AKT and MAPK signaling pathways. J Transl Med.

[3] Lu J, Zheng ZF, Wang W (2019). A novel TNM staging system for gastric cancer based on the metro-ticket paradigm: a comparative study with the AJCC-TNM staging system. Gastric Cancer.

[4] Jin Y, Qiu MZ, Wang DS (2013). Adjuvant chemotherapy for elderly patients with gastric cancer after D2 gastrectomy. PLoS One.

[5] Park JM, Kim JH, Park SS (2008). Prognostic factors and availability of D2 lymph node dissection for the patients with stage II gastric cancer: comparative analysis of subgroups in stage II. World J Surg.

[6] Sekido Y, Mukai M, Yamazaki M (2014). Occult neoplastic cells in lymph node sinuses and recurrence/metastasis of stage II/III gastric cancer. Oncol Lett.

[7] Noh SH, Park SR, Yang HK (2014). Adjuvant capecitabine plus oxaliplatin for gastric cancer after D2 gastrectomy (CLASSIC): 5-year follow-up of an open-label, randomised phase 3 trial. Lancet Oncol.

[8] Bang YJ, Kim YW, Yang HK (2012). Adjuvant capecitabine and oxaliplatin for gastric cancer after D2 gastrectomy (CLASSIC): a phase 3 open-label, randomised controlled trial. Lancet.

[9] Jiang Y, Li T, Liang X (2017). Association of Adjuvant Chemotherapy With Survival in Patients With Stage II or III Gastric Cancer. JAMA Surg.

[10] Sasako M, Sakuramoto S, Katai H (2011). Five-year outcomes of a randomized phase III trial comparing adjuvant chemotherapy with S-1 versus surgery alone in stage II or III gastric cancer. J Clin Oncol.

[11] Yoshikawa T, Terashima M, Mizusawa J (2019). Four courses versus eight courses of adjuvant S-1 for patients with stage II gastric cancer (JCOG1104 [OPAS-1]): an open-label, phase 3, non-inferiority, randomised trial. Lancet Gastroenterol Hepatol.

[12] Imamura T, Komatsu S, Ichikawa D (2015). Poor prognostic subgroup in T3N0 stage IIA gastric cancer, suggesting an indication for adjuvant chemotherapy. J Surg Oncol.

[13] Liebig C, Ayala G, Wilks J (2009). Perineural invasion is an independent predictor of outcome in colorectal cancer. J Clin Oncol.

[14] Tao Q, Zhu W, Zhao X (2020). Perineural invasion and postoperative adjuvant chemotherapy efficacy in patients with gastric cancer. Front Oncol.

[15] Koide N, Yamada T, Shibata R (2006). Establishment of perineural invasion models and analysis of gene expression revealed an invariant chain (CD74) as a possible molecule involved in perineural invasion in pancreatic cancer. Clin Cancer Res.

[16] Amin MB, Greene FL, Edge SB (2017). The Eighth Edition AJCC Cancer Staging Manual: Continuing to build a bridge from a population-based to a more "personalized" approach to cancer staging. CA Cancer J Clin.

[17] Ma J, Shen H, Kapesa L (2016). Lauren classification and individualized chemotherapy in gastric cancer. Oncol Lett.

[18] Zhang X, Liang H, Li Z (2021). Perioperative or postoperative adjuvant oxaliplatin with S-1 versus adjuvant oxaliplatin with capecitabine in patients with locally advanced gastric or gastro-oesophageal junction adenocarcinoma undergoing D2 gastrectomy (RESOLVE): an open-label, superiority and non-inferiority, phase 3 randomised controlled trial. Lancet Oncol.

[19] Yu J, Huang C, Sun Y (2019). Effect of laparoscopic vs open distal gastrectomy on 3-year disease-free survival in patients with locally advanced gastric cancer: the CLASS-01 Randomized Clinical Trial. JAMA.

[20] Yang MW, Tao LY, Jiang YS (2020). Perineural invasion reprograms the immune microenvironment through cholinergic signaling in pancreatic ductal adenocarcinoma. Cancer Res.

[21] Xia Q, Bai QR, Dong M (2015). Interaction between gastric carcinoma cells and Neural Cells Promotes Perineural Invasion by a Pathway Involving VCAM1. Dig Dis Sci.

[22] Jiang SH, Zhang S, Wang H (2022). Emerging experimental models for assessing perineural invasion in human cancers [J]. Cancer Lett.

[23] Poeschl EM, Pollheimer MJ, Kornprat P (2010). Perineural invasion: correlation with aggressive phenotype and independent prognostic variable in both colon and rectum cancer. J Clin Oncol.

[24] Zhao B, Lv W, Mei D (2020). Perineural invasion as a predictive factor for survival outcome in gastric cancer patients: a systematic review and meta-analysis. J Clin Pathol.

[25] Chen YF, Wang SY, Le PH (2022). Prognostic Significance of Perineural Invasion in Patients with Stage II/III Gastric Cancer Undergoing Radical Surgery. J Pers Med.

[26] Zheng X, Wu Y, Zheng L (2021). Disease-specific survival of AJCC 8th stage II gastric cancer patients after D2 gastrectomy. Front Oncol.

[27] Japanese Gastric Cancer A (2017). Japanese gastric cancer treatment guidelines 2014 (ver. 4). Gastric Cancer.

[28] Nakamura K, Hatakeyama K, Furukawa K (2020). Prediction of S-1 adjuvant chemotherapy benefit in Stage II/III gastric cancer treatment based on comprehensive gene expression analysis. Gastric Cancer.

[29] Deng ZJ, Lu J, Nie RC (2022). Indications for Adjuvant Chemotherapy in Stage II Gastric Cancer After D2 Gastrectomy–A Chinese Multicenter Study. Ann Surg Oncol.

[30] Huang JB, Lu J, Wu D (2021). Is Adjuvant Chemotherapy Beneficial to All Patients With pT3N0M0 Stage Gastric Cancer?. Front Oncol.

