# Supplementary material for: Implication of perineural invasion in patients with stage II gastric cancer
Source: World J Surg Oncol. 2023 Nov 29;21:372. doi: 10.1186/s12957-023-03236-x (PMC10685670; doi:10.1186/s12957-023-03236-x)
Supplement: Supplementary file 1 — Additional file 1: Supplementary Figure 1. Survival curve analysis showed whether patients with or without PNI received adjuvant chemotherapy (A) or without adjuvant chemotherapy (B). Supplementary Table1. Recurrence pattern of Stage II GC patients with PNI. Supplementary Table2. T stage and Lymph node metastasis of Stage II GC patients with or without PNI [file 12957_2023_3236_MOESM1_ESM.docx]

**Supplementary Material**


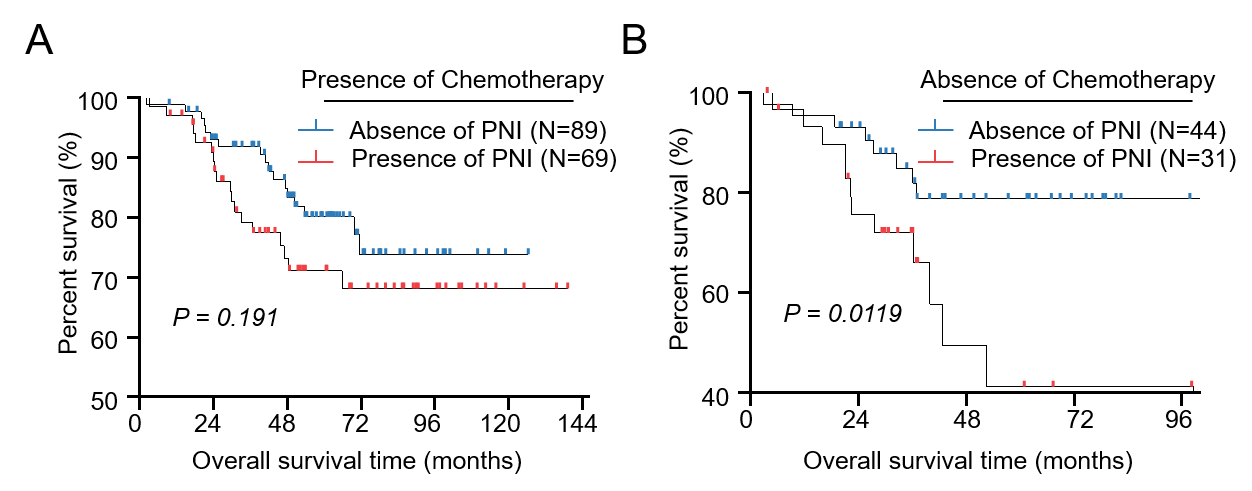


Supplementary Figure 1. Survival curve analysis showed whether patients with or without PNI received adjuvant chemotherapy (A) or without adjuvant chemotherapy (B).

**Supplementary Table1**

Recurrence pattern of Stage II GC patients with PNI

|  |  |  | PNI | |  |
| --- | --- | --- | --- | --- | --- |
| Characteristics |  | Overall (N=65) | Absence (N=31) | Presence (N=34) | p value |
| Recurrence events (%) | Liver | 4 (6.15) | 3 (9.68) | 1 (2.94) | **0.038** |
|  | Local | 3 (4.62) | 2 (6.45) | 1 (2.94) |  |
|  | Multiple sites | 4 (6.15) | 2 (6.45) | 2 (5.88) |  |
|  | Other or uncertain sites | 31 (47.69) | 19 (61.29) | 12 (35.29) |  |
|  | Peritoneum | 23 (35.38) | 5 (16.13) | 18 (52.94) |  |
| DFS (median [IQR]) |  | 25.667 [18.767, 41.967] | 27.400 [20.450, 45.350] | 24.633 [17.725, 36.517] | 0.415 |
| DFS | <24 month | 28 (43.08) | 12 (38.71) | 16 (47.06) | 0.669 |
|  | ≥24 month | 37 (56.92) | 19 (61.29) | 18 (52.94) |  |

IQR,interquartile range.

**Supplementary Table2**

T stage and Lymph node metastasis of Stage II GC patients with or without PNI

|  | PNI | |
| --- | --- | --- |
| Characteristics | Absence (N=133) | Presence (N=100) |
| T1/Lymph node metastasis Presence | 9 (6.77) | 0 (0) |
| T2/Lymph node metastasis Presence | 28 (21.05) | 5 (5) |
| T3/Lymph node metastasis Absence | 59 (44.36) | 50 (50) |
| T3/Lymph node metastasis Presence | 36 (27.07) | 36 (36) |
| T4a/Lymph node metastasis Absence | 1 (0.75) | 9 (9) |
